# Cardiomyogenic differentiation is fine-tuned by differential mRNA association with polysomes

**DOI:** 10.1186/s12864-019-5550-3

**Published:** 2019-03-15

**Authors:** Isabela Tiemy Pereira, Lucia Spangenberg, Anny Waloski Robert, Rocío Amorín, Marco Augusto Stimamiglio, Hugo Naya, Bruno Dallagiovanna

**Affiliations:** 1 Basic Stem-cell Biology Laboratory, Instituto Carlos Chagas - FIOCRUZ-PR, Rua Professor Algacyr Munhoz Mader, 3775, Curitiba, PR 81.350-010 Brazil; 2 Bioinformatics Unit, Institut Pasteur de Montevideo, Mataojo 2020, 11400 Montevideo, Uruguay

**Keywords:** Cardiomyogenesis, hESC-derived cardiomyocytes, Polysome profiling, Post-transcriptional regulation

## Abstract

**Background:**

Cardiac cell fate specification occurs through progressive steps, and its gene expression regulation features are still being defined. There has been an increasing interest in understanding the coordination between transcription and post-transcriptional regulation during the differentiation processes. Here, we took advantage of the polysome profiling technique to isolate and high-throughput sequence ribosome-free and polysome-bound RNAs during cardiomyogenesis.

**Results:**

We showed that polysome-bound RNAs exhibit the cardiomyogenic commitment gene expression and that mesoderm-to-cardiac progenitor stages are strongly regulated. Additionally, we compared ribosome-free and polysome-bound RNAs and found that the post-transcriptional regulation vastly contributes to cardiac phenotype determination, including RNA recruitment to and dissociation from ribosomes. Moreover, we found that protein synthesis is decreased in cardiomyocytes compared to human embryonic stem-cells (hESCs), possibly due to the down-regulation of translation-related genes.

**Conclusions:**

Our data provided a powerful tool to investigate genes potentially controlled by post-transcriptional mechanisms during the cardiac differentiation of hESC. This work could prospect fundamental tools to develop new therapy and research approaches.

**Electronic supplementary material:**

The online version of this article (10.1186/s12864-019-5550-3) contains supplementary material, which is available to authorized users.

## Background

Recent approaches regarding hESCs differentiation made it possible to mimic features of developmental biology and address the key events that regulate early lineage commitment [1]. Cardiomyocytes have been derived from hESCs as an in vitro model to study cardiomyogenesis and as an attempt to produce clinically relevant cell populations [2–5]. Modeling congenital abnormalities of the heart or testing the cardiac toxicity of new drugs strengthen a particular interest in deriving cardiovascular lineages from pluripotent stem cells [2]. Additionally, in vitro cardiomyocyte generation and promotion of an endogenous regenerative capacity offers new therapeutic strategies to replace heart tissue damaged by age or disease [6, 7]. Activation of a specific genetic program is essential to drive cells into the cell type of interest and contribute to these challenging goals. Cardiac tissue formation is controlled by sequential gene regulatory steps that define specialized cell fates [8], although understanding of molecular signatures of intermediate differentiation states of the cardiomyogenic lineage are still lacking.

Gene expression regulation acts at both transcriptional and post-transcriptional levels. There has been an increasing interest in understanding how these regulatory mechanisms interact and coordinate along the differentiation processes [9]. Large-scale studies help to uncover the complex and dynamic multi-layered regulation involving chromatin modifications, transcriptional, and post-transcriptional networks that control the reconfiguration of ESC gene expression program when cells are stimulated to differentiate [10, 11]. Genome-wide expression profiling, using microarrays or RNA-seq analysis, has provided the possibility to investigate transcriptional changes during cardiomyogenic differentiation [12–17]. However, most of these studies were centered at total transcriptome analysis. Since the cellular abundance of proteins is also controlled at the translation level [18], the most-reliable gene expression patterns cannot be based solely on total RNA analysis.

The role of translational control as a key regulatory node in gene expression during development and disease is still being established. Actively translated mRNAs are predicted to be associated with many ribosomes, which form large complexes called polysomes [19]. These complexes define the translatome, the subpopulation of mRNAs that are engaged with the translation machinery. Methods to infer the translatome have helped to expand our knowledge of protein synthesis control and revealed post-transcriptional mechanisms involved in cell fate commitment [20–22]. A classical technique called polysome profiling has been used to assess the mRNAs bound to ribosomes through ultracentrifugation in a sucrose gradient, and further identify them by high-throughput methods [23–25]. Previously, we used polysome profiling to study the fate of human adipose stem cells (hASCs) and their commitment to adipogenesis [25, 26]. We showed that 60% of the genes, which were differentially expressed after 72 h of differentiation induction, were controlled by post-transcriptional regulation.

Here, we used polysome profiling to assess the ribosome-free and polysome-bound mRNAs during the developmental steps of cardiomyogenic commitment and investigate genes potentially controlled by post-transcriptional mechanisms. Our results showed that 60–80% of differentially expressed genes (DEG) showed some degree of post-transcriptional regulation, suggesting its crucial role in shaping of the developmental pathways. Additionally, committed cells showed decreased protein synthesis rates compared to hESC.

## Results

### Polysome profiling during hESCs cardiomyogenic differentiation

**Fig. 1:**
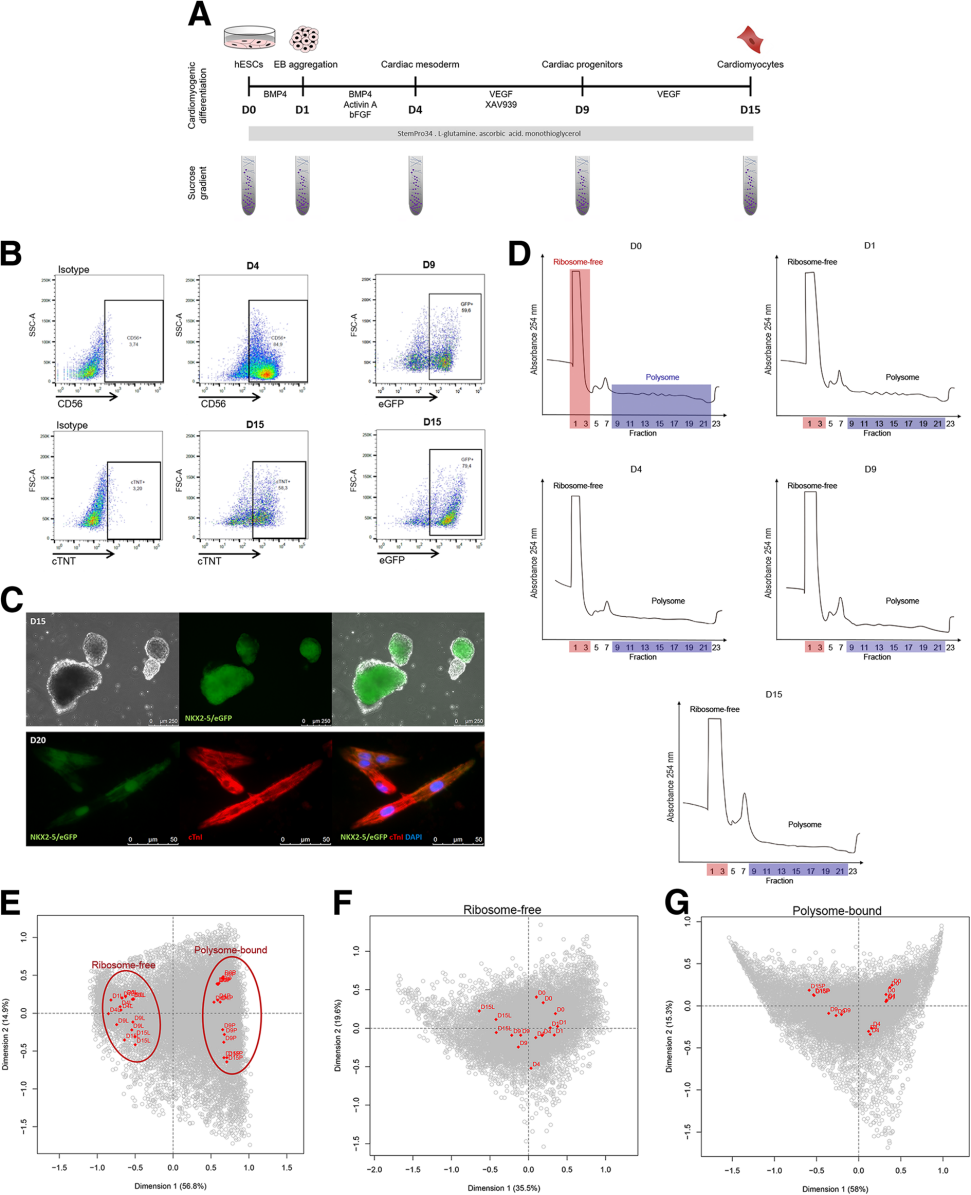
Polysome profiling of hESC during cardiomyogenic differentiation. (**a**) Schematic representation of cardiomyogenic differentiation protocol indicating developmental stages and timing of sample collection. (**b**) Flow cytometry analysis of D4 (CD56), D9 (eGFP) or D15 (cTnT and eGFP) differentiating cells. Representative dot plots (*n* = 3). (**c**) Representative images of EBs during differentiation showing NKX2–5/eGFP expression on D15 (top panel) and immunostaining of cTNI on D20 (bottom panel). Cells were co-stained with DAPI to visualize the nucleus. (**d**) Representative polysome profile obtained by sucrose gradient of cells at distinct time-points of differentiation (n = 3). Ribosome-free (red) and polysome (blue) fractions are indicated. Correspondence analysis (COA) of (**e**) all sequenced samples (total 30 samples), (**f**) ribosome-free and (**g**) polysome-bound samples at days D0, D1, D4, D9 and D15 (n = 3). See also Additional file 2: Figure S1

The hES-NKX2–5^eGFP/w^ reporter human embryonic stem cell (hESC) line [27] was used to derive cardiomyocytes using a developmentally staged protocol [2, 28] that includes the induction of a cardiac mesoderm population on days 3 and 4 and a NKX2–5^+^/cTNT^+^ population by day 15 (Fig. 1a and b). Cardiomyogenesis progression was followed by flow cytometry using CD56 as a mesoderm marker [29] on day 4 (cut off < 40%) and NKX2–5/eGFP expression on day 9 as a cardiac progenitor marker (cut off < 50%) (Fig. 1b). Beating clusters were observed after 10 days of differentiation (Fig. 1c and Online Additional file 1: Video S1), yielding a population of 50–60% cTNT^+^ cardiomyocytes on day 15 (D15) (Fig. 1b). At day 20, cTNI immunostaining showed the striations characteristic of sarcomere structures (Fig. 1c).

To investigate the differential association of mRNAs with polysomes and, therefore, post-transcriptional changes in gene expression during cardiac differentiation, we first performed polysome profiling on days D0, D1, D4, D9 and D15, which represent pluripotency, embryoid body (EB) aggregation, cardiac mesoderm, cardiac progenitor and cardiomyocyte stages, respectively (Fig. 1a). After 10 min of cycloheximide treatment, active ribosomes got arrested with associated RNAs and we analyzed cellular extracts by ultracentrifugation in a sucrose gradient (Fig. 1d). Differential density throughout the gradient allowed the isolation of ribosome-free (fractions 1–3) and polysome-bound (fractions 9–22) RNAs. Based on the polysome profile, pooled ribosome-free and polysome-bound RNA fractions were sequenced using the Illumina platform, yielding nearly 30 million reads for each sample. Approximately 70–80% of the reads were mapped onto the reference genome (GRCh38), and more than 17,000 genes were detected in each type of fraction (Additional file 2: Figure S1). As a control, D15 cells were also treated with puromycin to disassemble the polysomes and cardiomyocyte markers were evaluated by qPCR (Additional file 2: Figure S2).

Correspondence analysis (COA) showed that samples were grouped according to the type of RNA fraction (ribosome-free vs. polysome-bound) (Fig. 1e) and according to the day of differentiation (D0, D1, D4, D9 and D15) (Fig. 1f and g). Polysome-bound samples showed more distinct groups relative to the day of differentiation, indicating high similarity between translated genes in experimental replicates (Fig. 1g). On the other hand, ribosome-free samples showed more dispersion and less similarity between the replicates.

### The translatome delineates cardiomyogenic gene expression

**Fig. 2:**
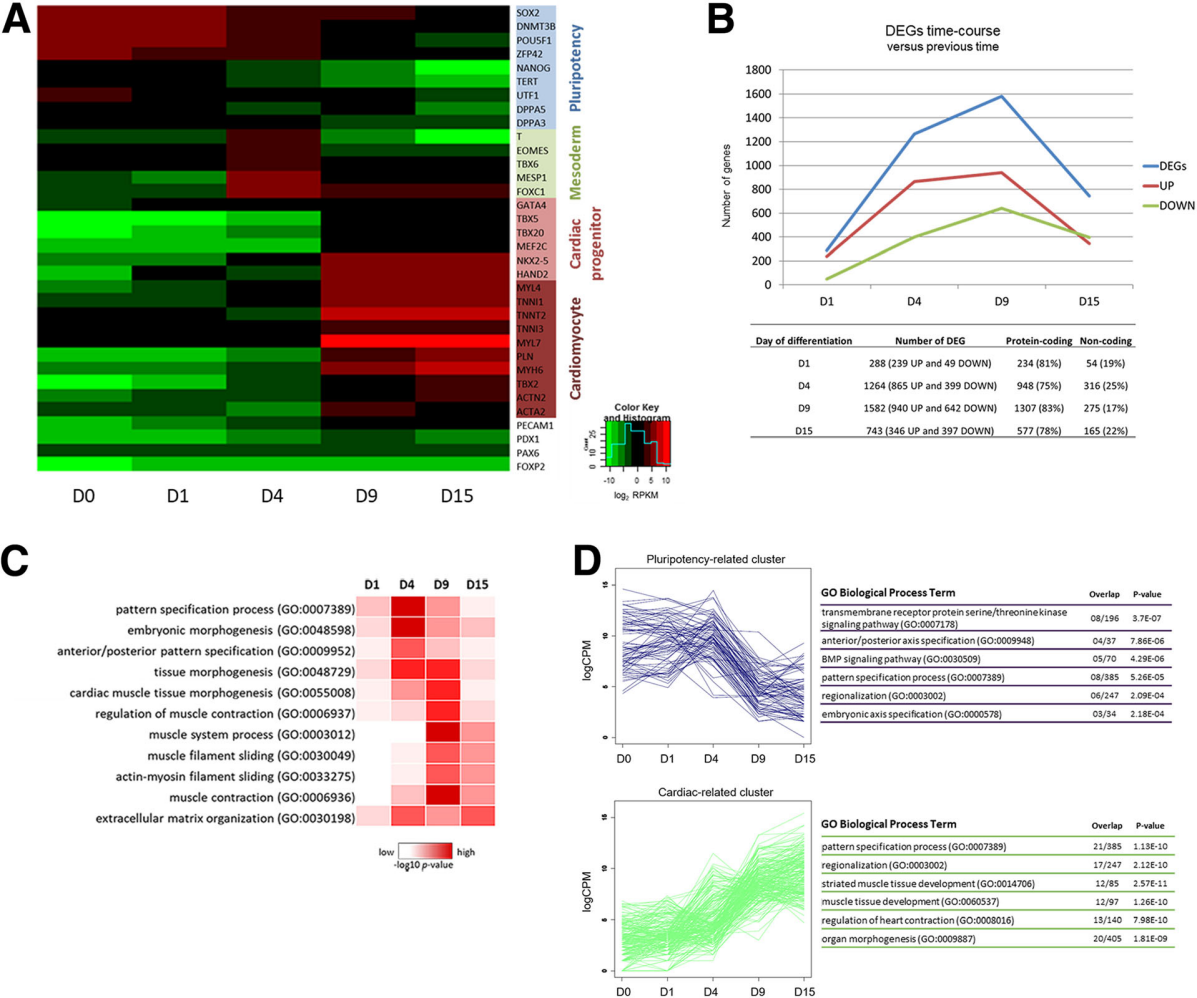
Polysome-bound RNA-seq revealed massive regulation of developmental genes. (**a**) Expression of lineage marker genes quantitated by -log2 RPKM. Stage markers are color-coded. Blue: pluripotency. Green: mesoderm. Light red: cardiac progenitors. Dark red: cardiomyocytes. Uncolored: endoderm and ectoderm. (**b**) Numbers of differentially expressed genes at each differentiation time-point, compared to the preceding time-point (FDR < 0.05, − 2 > logFC> 2). Numbers of protein-coding and non-coding genes are also shown (bottom panel). (**c**) Gene Ontology analysis of EnrichR Biological Process (BP) enriched terms for up-regulated (FDR < 0.05, logFC> 2) genes during cardiomyocyte differentiation when compared to each preceding time-point. (**d**) Gene expression pattern clusters of pluripotency (top) and cardiac (bottom) related genes and their EnrichR BP enriched terms. Six terms with lower *p*-values are shown. See also Additional file 2: Figures S4 and S5

During heart development, temporal gene expression changes occur to define each step of cardiogenic commitment [6, 30]. RPKM values (reads per kilobase per million mapped reads) for polysome-bound RNA fraction showed the expression levels of known lineage-specific genes throughout cardiac differentiation (Fig. 2a) which were confirmed by qPCR (Additional file 2: Figures S2 and S3). Pluripotency marker genes, including *SOX2, POU5F*1 (OCT4) and *NANOG*, were expressed at higher levels on D0 and D1 and down-regulated at following time-points. The mesoderm marker genes *T* and *EOMES*, and early cardiac gene *MESP1* were highly specifically expressed on D4. Expression of cardiac-related genes such as *GATA4, NKX2–5* and *TBX5 *as well as cardiomyocyte-specific sarcomeric genes *TNNI, TNNT, MYL*7 and *MYH*6 were observed from D9 to D15. In contrast, the endoderm (*PECAM1* and *PDX1*) and ectoderm (*PAX6* and *FOXP2*) marker genes did not change their polysome association during cardiomyogenic differentiation (Fig. 2a).

Comparisons between each differentiation time-point and the preceding time-point, considering an overall FDR of ≤0.05 and − 2 ≥ logFC ≥2, identified differentially expressed genes (DEGs) in polysome-bound RNA fractions (Fig. 2b, data available in Additional file 3). Aggregation of embryoid bodies during the first 24 h of differentiation induced differential expression of 288 genes. Mesoderm commitment from D1 to D4 showed 1264 DEGs, and cardiac progenitor progression to D9 showed 1582 DEGs. The final step of differentiation analysis on D15 showed 743 DEGs compared to D9. In general, the majority of DEGs were up-regulated, except for D15 compared to D9, where the number of down-regulated genes was slightly higher than up-regulated genes (Fig. 2b). Similar numbers of genes and patterns of up- and down-regulated genes were shown in the ribosome-free samples (Additional file 2: Figure S4A and Additional file 4). Regarding the protein-coding and non-coding genes in polysome-bound samples, approximately 20% of DEGs in each time-point analysis are annotated as non-coding RNAs, in which 39% correspond to “lincRNA”, 22% to “antisense” and 18% to “processed pseudogene” RNAs (Additional file 2: Figure S4B).

Gene Ontology (GO) analysis of polysome-bound up-regulated DEGs (FDR ≤ 0.05 and logFC ≥2) revealed developmental and cardiac-related “biological process” (BP) during cardiac differentiation (Fig. 2c). BP terms *p*-values (−log10) of DEGs for each time-point compared to the preceding one are represented in Fig. 2c heatmap. “Cardiac muscle tissue morphogenesis” and “regulation of muscle contraction” are highly enriched on D9 (compared to D4) and only slightly enriched on D15 (compared to D9), indicating that on D9, most cardiac characteristics are already committed. This pattern is similar for “muscle system process” and “muscle contraction”. “Extracellular matrix organization” seems to have important roles in two distinct phases: mesoderm commitment (D4) and cardiomyocyte final differentiation (D15) (Fig. 2c and Additional file 2: Figure S5).

To assess more information about variations in gene expression during cardiac differentiation, we performed gene clustering using logCPM (counts per million mapped reads). Distinct pattern expression groups were shown: genes with decreased expression during the differentiation, called pluripotency-related cluster and enriched in early developmental BP terms, such as “anterior/posterior axis specification” and “BMP signaling pathway”; and genes with increased expression during cardiac differentiation, called cardiac-related cluster and enriched in lineage specific commitment terms, such as “muscle tissue development” and “regulation of heart contraction” (Fig. 2d).

### Strong gene expression coordination is observed during mesoderm-to-cardiac progenitor commitment

**Fig. 3:**
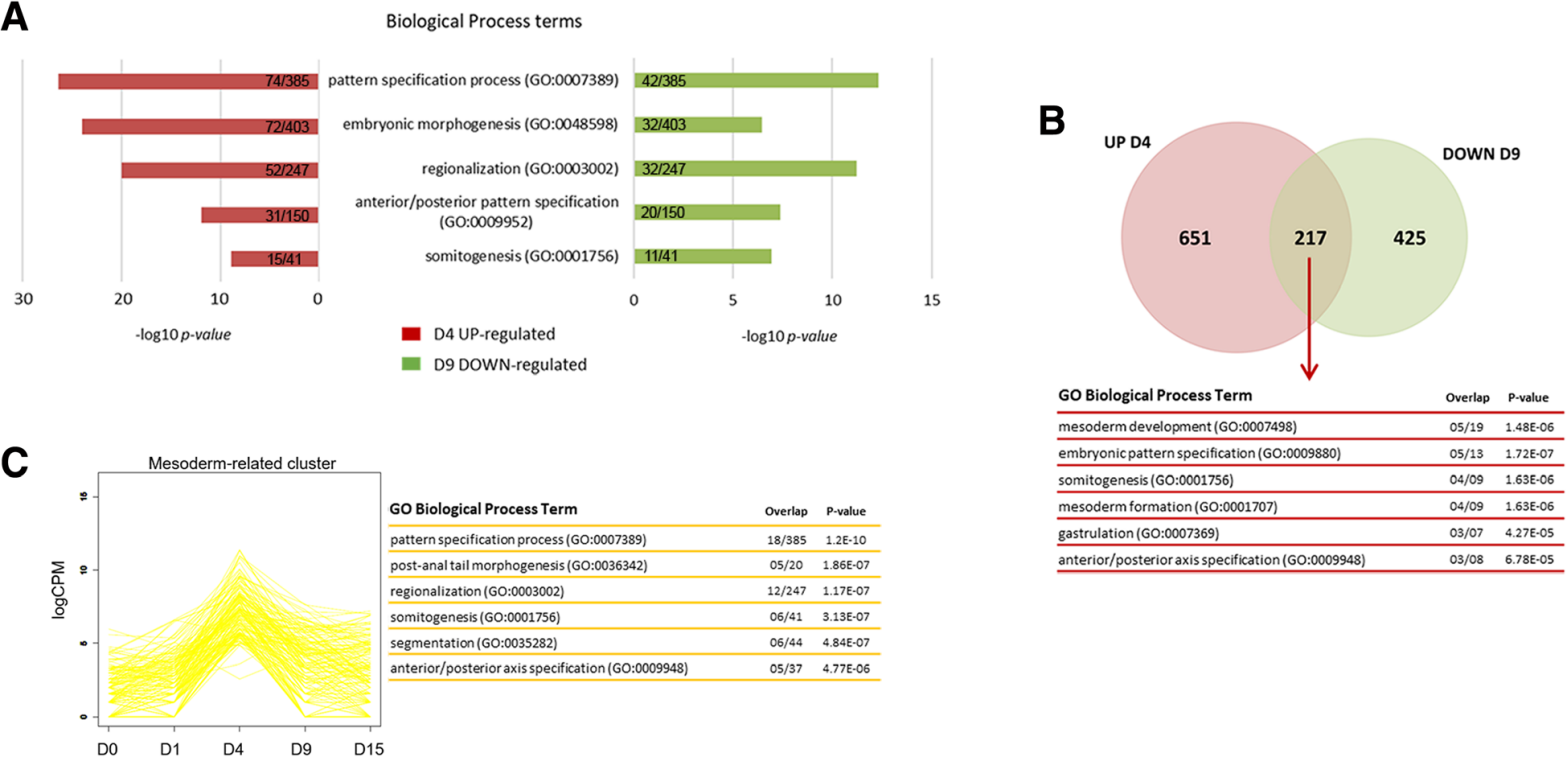
Mesoderm and cardiac progenitor commitment gene expression. (**a**) GO EnrichR BP enriched terms for D4 up-regulated (FDR < 0.05, logFC> 2) and D9 down-regulated genes (FDR < 0.05, logFC<− 2). Overlap is shown inside the bars for each term in each condition. (**b**) Venn diagram and GO EnrichR BP enriched terms of common D4 up-regulated (FDR < 0.05, logFC> 2) and D9 down-regulated genes (FDR < 0.05, logFC<− 2). Six terms with lower p-values are shown. (**c**) Gene expression pattern cluster of mesoderm-related genes and their GO EnrichR BP enriched terms. Six terms with lower p-values are shown

When considering polysome-bound RNAs, the largest gene expression variation showing 1582 DEGs occurred during the D4 to D9 shift, which represents mesoderm-to-cardiac progenitor commitment (Fig. 2b). GO analysis revealed that some of D4 up-regulated BP terms were also enriched in the D9 down-regulated analysis (Fig. 3a and Additional file 2: Figure S5), such as “pattern specification process” and “embryonic morphogenesis”. These findings suggest a crucial gene expression regulation at this stage. Comparisons between D4 up-regulated and D9 down-regulated genes showed 217 in common, which are related to “mesoderm development” and “embryonic pattern specification” BP terms (Fig. 3b).

Moreover, an expression gene clustering pattern showed a distinct group of genes highly and specifically expressed on D4 (Fig. 3c). Those genes are called mesoderm-related genes and are enriched on developmental processes, such as “pattern specification process”, “regionalization” and “somitogenesis”. Altogether, these results indicate that the developmental progress of mesoderm-to-cardiac progenitor is carefully regulated and can be assessed by polysome-bound RNA analysis.

### Cardiac commitment is intensely tuned by differential mRNA association with polysomes

**Fig. 4:**
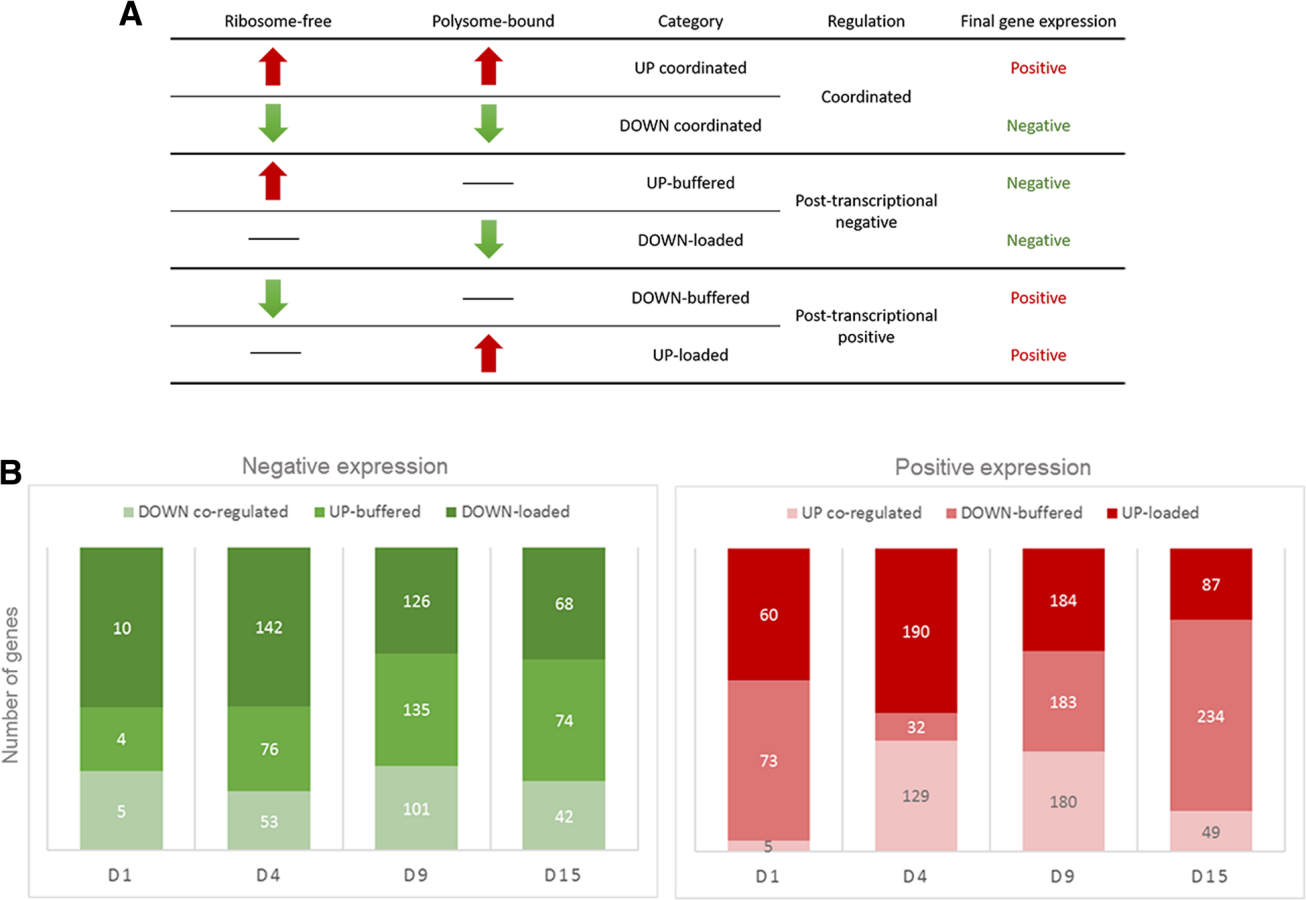
Post-transcriptionally regulated genes during cardiomyogenesis. (**a**) Gene categories based on the ribosome-free and polysome-bound comparison analysis. (**b**) Number of DEGs (FDR < 0.05, − 2 > logFC> 2, RPKM> 1) classified at the gene regulation categories. See also Additional file 2: Figure S6

Gene expression fluctuations could be a consequence of coordination or lack thereof between transcription and translation rate changes. To assess the post-transcriptional regulation during the cardiomyogenic differentiation, we performed the two step-analysis: (1) DEGs were identified through the comparison between each differentiation time-point and the preceding time-point, considering an overall FDR ≤ 0.05, − 2 ≥ logFC ≥2 and RPKM > 1 on ribosome-free or polysome-bound samples (Additional files 3 and 4, respectively); and (2) ribosome-free DEGs and polysome-bound DEGs were compared and classified according to the following categories (Additional file 5). Genes that were up- or down-regulated in both fractions were labeled “up-coordinated” or “down-coordinated”. Transcripts that were up-regulated in ribosome-free fraction but might be neutralized by post-transcriptional mechanisms were labeled “up-buffered”, or in the opposite case, as “down-buffered”. Moreover, genes showing that their polysome association was increased or decreased, were labeled “up-loaded” or “down-loaded”, respectively (Fig. 4a).

We used the coordinated, buffered and loaded classification of DEGs and included one more label category considering the final gene expression as coordinated, post-transcriptional positive or negative regulation (Fig. 4a). Therefore, when compared to the preceding time-point, genes up- or down-coordinated were labeled being under coordinated regulation, genes up-buffered and down-loaded under post-transcriptional negative regulation and genes down-buffered and up-loaded under post-transcriptional positive regulation. Approximately 60–80% of DEGs showed one or another kind of post-transcriptional regulation, positive or negative, suggesting a crucial role of this level of gene expression control (Fig. 4b). Interestingly, during the initial steps of differentiation (D0-D1 and D1-D4), there was a prevalence of up- (10/19 and 142/271) or down-loaded (60/138 and 190/351) genes, suggesting a strong post-transcriptional regulation at these stages. During cardiac progenitor commitment on D9, numbers of coordinated, buffered and loaded genes were similar, either on positive or negative regulation. On the other hand, between D9 and D15 most genes were classified as buffered, in particular, on positive regulation (234/370), indicating that transcriptional variations might be controlled by post-transcriptional mechanisms. These results corroborate our previous findings showing that most of cardiac characteristics are already committed on D9 (Fig. 2).

Considering that Gene Ontology-annotated genes are usually protein-coding, we filtered our data and performed GO analysis using only protein-coding genes. Analysis of coordinately regulated genes showed well-established pathways during hESC differentiation, such as the Reactome pathway terms “POU5F1 (OCT4), SOX2, NANOG repress genes related to differentiation”, “Transcriptional regulation of pluripotent stem cells” and “Developmental Biology”, which were upregulated on D1 and D4 and down-regulated later (D9 and D15) (Additional file 2: Figure S6). Additionally, the cardiac-related pathway “Muscle contraction” and cardiac-specific pathway “Cardiac conduction” were coordinately up-regulated at D9 and D15 time-points (Additional file 2: Figure S6).

**Fig. 5:**
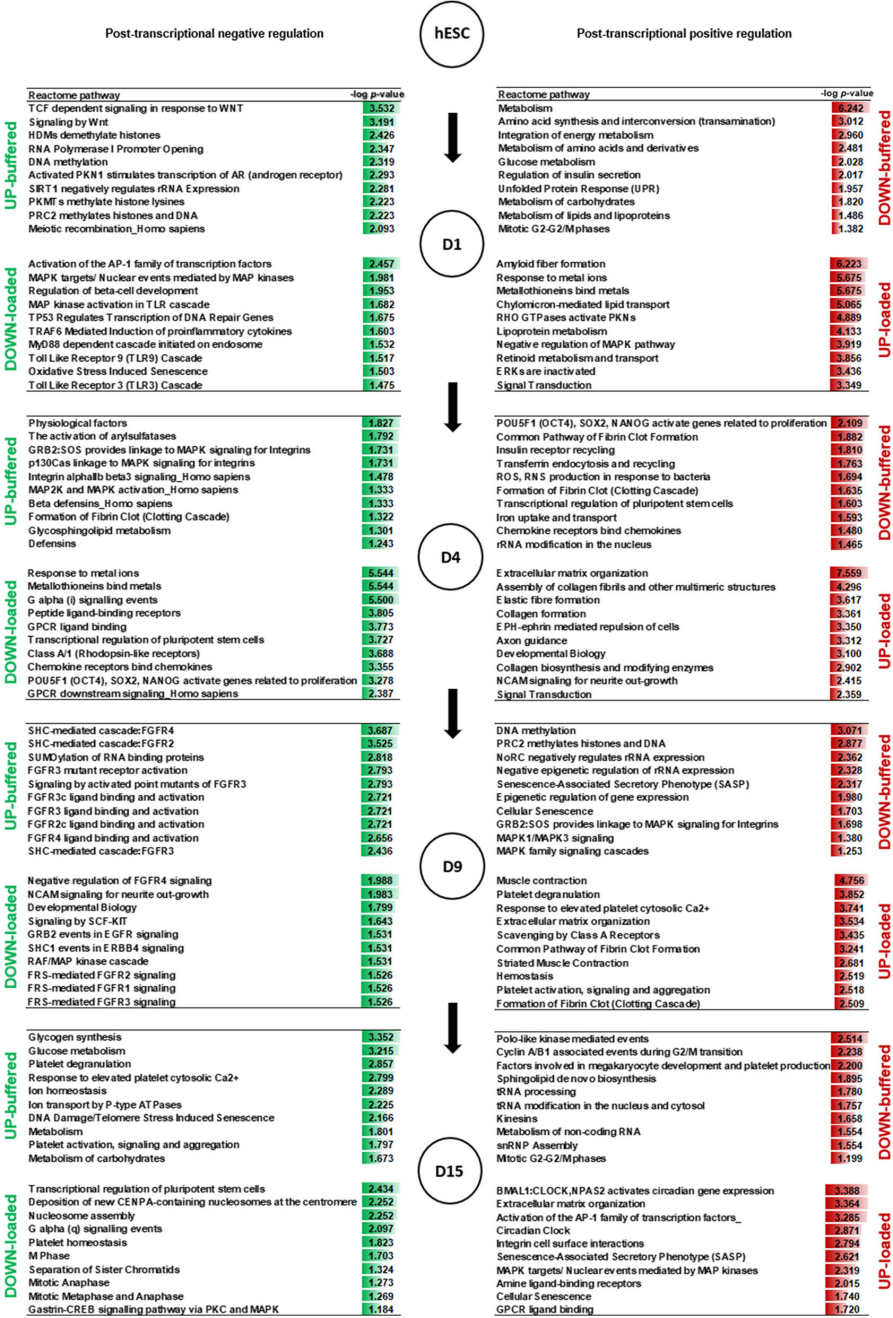
Post-transcriptionally regulated genes during cardiomyogenic differentiation were involved in diverse biological processes. GO Reactome pathways enriched terms for up- (FDR < 0.05, logFC> 2, RPKM> 1) and down- (FDR < 0.05, logFC<− 2, RPKM> 1) buffered and loaded genes. Ten terms with lower p-values are shown

Genes classified as buffered or loaded showed enriched pathway terms with a diversity of biological processes (Fig. 5). The Reactome pathways “Developmental Biology” and “NCAM signaling for neurite out-growth” were up-loaded on D4 and down-loaded on D9, once more suggesting the critical regulation at this stage. For instance, developmental-related genes, such as *NOTUM, CER1* and *SOX17*, appeared as up-loaded on D4 and down-loaded on D9 (Additional file 5 and Additional file 2: Figure S7A), indicating the polysomal loading regulation. The “M phase” and “Mitotic Metaphase and Anaphase” terms were shown as down-loaded on D15, while “Cyclin A/B1 associated events during G2/M transition” as down-buffered, indicating the fine adjustment of cell cycle during differentiation. The E2F Transcription Factor 1 plays a crucial role in the control of cell cycle [31] and was shown as involved in myoblast proliferation and differentiation through the auto-regulation loop with miR-20a-5p and miR-20b-5p [32]. E2F1 gene appeared less associated to polysomes on D9 (FDR < 0.05, logFC − 1.73, not included on down-loaded group), and down-buffered on D15, illustrating an initial polysomal dissociation followed by transcriptional down regulation of this gene (Additional files 3 and 4).

We have previously shown that “Extracellular matrix organization” seems to have an important role during differentiation (Fig. 2c), and it is probably also being post-transcriptionally regulated once it showed as enriched for up-loaded genes on D4, D9 and D15 (Fig. 5). Between the D15 up-loaded genes are *LUM, COL6A3* and *COL3A1 *(Additional file 5). *COL3A1* was already shown as post-transcriptionally regulated by the interaction of the heterogeneous nuclear ribonucleoprotein (hnRNP) A1 mRNA-binding protein with its 3′-UTR [33]. Interestingly, the cardiac-specific *NKX2–5* gene was shown as up-buffered on D4, suggesting that its RNA was transcribed but not translated yet. On D9, this gene was shown as up-coordinated, suggesting, in this case, its transcription and translation (Additional file 2 and Additional file 5: Figure S7B). In addition, other crucial cardiac transcription factors, such as *MEF2A* and *TBX5 *appeared as up-loaded on D9, indicating their polysome-bounding increase on this stage (Additional file 5 and Additional file 2: Figure S7C).

**Fig. 6:**
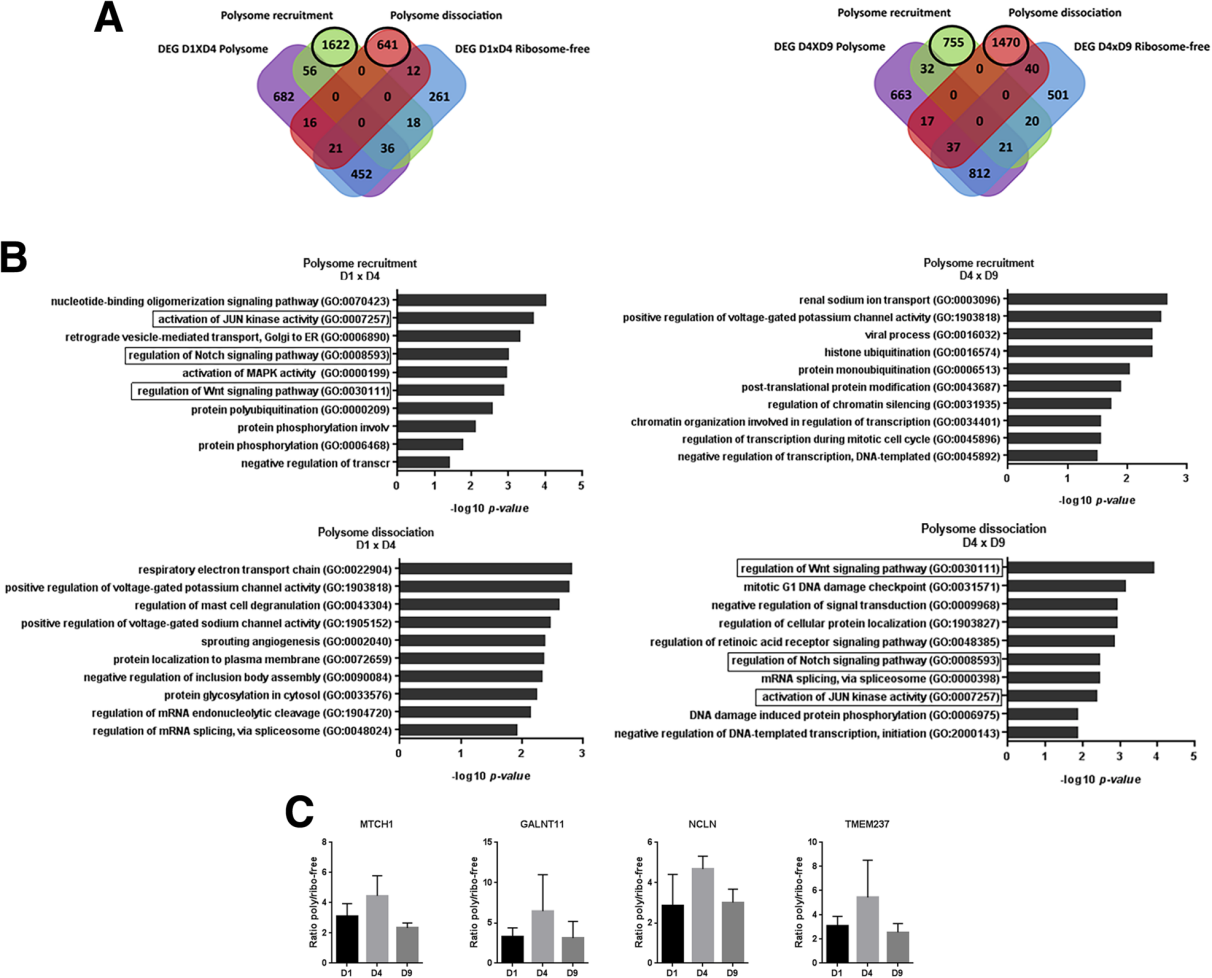
Non-DEGs showed differences on polysome recruitment and dissociation on D1 vs. D4 and D4 vs. D9. (**a**) Venn diagram and (**b**) GO EnrichR BP enriched terms of polysome recruitment (FDR < 0.05, logFC> 2) or dissociation (FDR < 0.05, logFC<− 2) for non-DEG based on polysome/ribosome-free ratio. (**c**) Polysome/ribosome-free ratio variation of the Notch and Wnt pathways genes during cardiomyocyte differentiation (FDR < 0.05, − 2 > logFC> 2). See also Additional file 2: Figure S8

To further characterize how recruitment to and dissociation from ribosomes influence gene expression, we performed polysome/ribosome-free ratio analysis using RPKM values. The results showed genes affected by polysome recruitment (FDR ≤ 0.05, logFC ≥2) or dissociation (FDR ≤ 0.05, − 2 ≥ logFC) during cardiomyocyte differentiation (Additional file 6). Some of them are also DEGs in polysome-bound or ribosome-free fractions, but most are not differentially expressed (Fig. 6a and Additional file 2: Figure S8). GO analysis for these non-differentially expressed and ratio-variated genes showed a variety of BP terms (Fig. 6b and Additional file 2: Figure S8). For instance, the development-related pathways JUN, Wnt and Notch were strongly regulated between D1, D4 and D9, were recruited from D1 to D4 and dissociated from D4 to D9 (Fig. 6b). The polysome/ribosome-free ratio of the JUN, Wnt and Notch pathway genes *MTCH1, GALNT11, NCLN* and *TMEM237* were plotted to visualize the variations on D1, D4 and D9 as an example (Fig. 6c).

### mRNA loading into polysomes fine-tunes crucial processes during hESC cardiomyogenesis

**Fig. 7:**
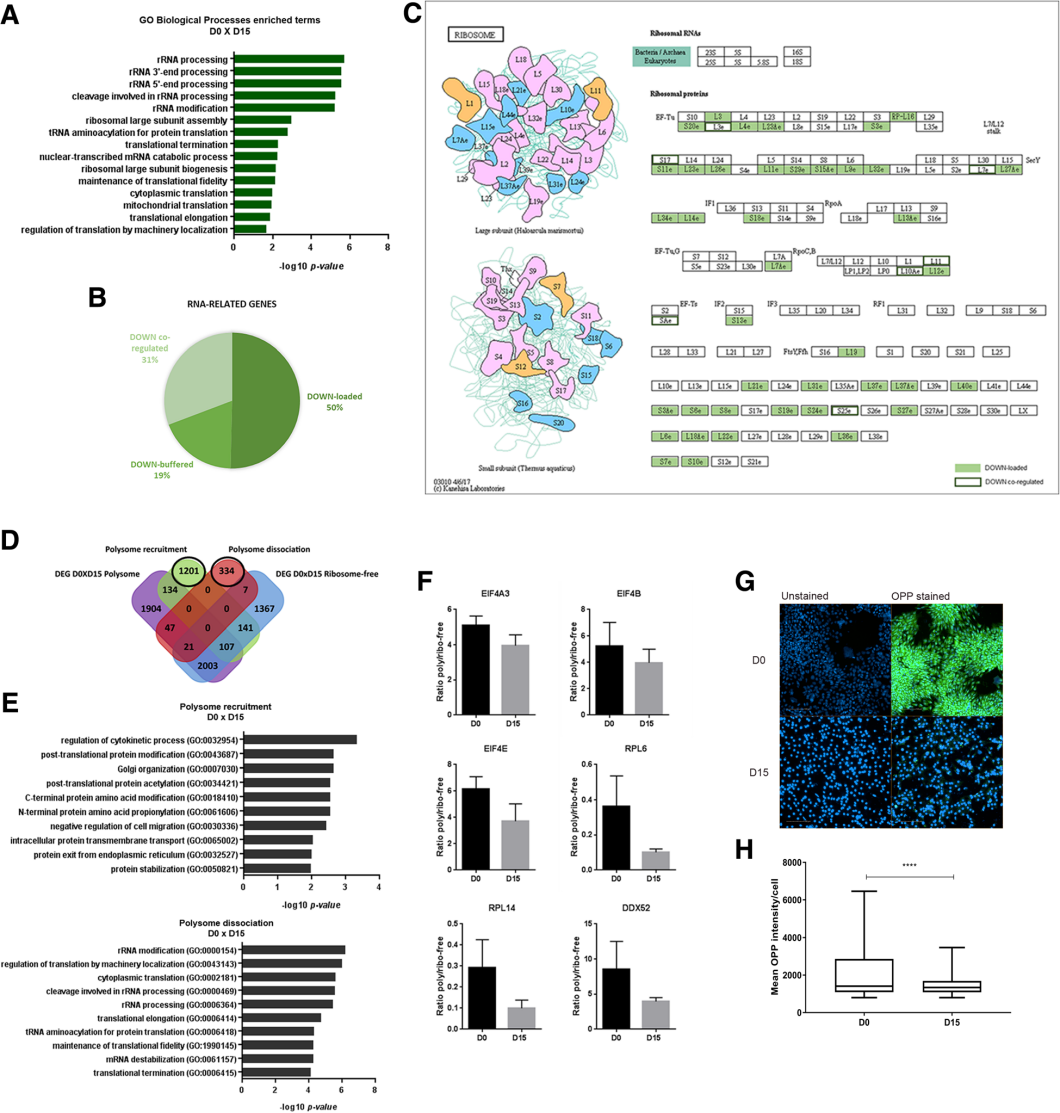
Cardiomyocytes (D15) showed down-regulation of translation and RNA processing genes. (**a**) GO EnrichR BP RNA-related terms enriched for D15 down-regulated genes (FDR < 0.05, logFC<− 1) compared to hESC (D0). (**b**) Genes classified on RNA-related BP terms (**a**) according to the co-regulated, buffered and loaded classification as indicated. (**c**) KEGG pathway analysis of D15 down-regulated ribosomal proteins. Down-loaded proteins showed in solid color, down co-regulated proteins showed as a green outline. (**d**) Venn diagram and (**e**) GO EnrichR BP enriched terms of polysome recruitment (FDR < 0.05, logFC> 2) or dissociation (FDR < 0.05, logFC<− 2) for non-DEG based on polysome/ribosome-free ratio of D0 vs. D15. (**f**) Translation genes showed polysome/ribosome-free ratio decreasing on D15 (FDR < 0.05, logFC<− 2). (**g**) Representative images of D0 and D15 cells cultured with OPP to stain nascent proteins. (**h**) Quantification of Alexa488 fluorescence intensity (OPP incorporation) at the cytoplasm region around the nucleus. For each condition (D0 and D15), 1400 cells were randomly chosen for intensity analysis. Statistical analysis was performed using the Mann-Whitney test (nonparametric t test). ****p < 0.0001

To better understand the changes in translation between hESC and cardiomyocytes, we performed Gene Ontology (GO) analysis with DEGs of D0 (hESC) vs. D15 (cardiomyocytes), considering FDR ≤ 0.05, − 1 ≥ logFC ≥1 and ribosome-free and polysome-bound data combined (Additional file 7). Genes down-regulated on D15 were enriched in Biological Process (BP) terms such as “rRNA processing”, “tRNA aminoacylation for protein translation” and “cytoplasmic translation”, which were grouped as “RNA-related terms” (Fig. 7a). Combining the genes annotated in this group (143 genes down-regulated on D15) and analyzing their post-transcriptional regulation classification, 44 (30.7%) of them were down-coordinated and 72 (50.3%) were down-loaded (Fig. 7b and Additional file 7). In addition, many ribosomal proteins were shown down-regulated on D15, predominantly down-loaded (90.9%) (Fig. 7c). Other translation machinery proteins were also down-regulated after cardiomyocyte commitment, for instance, the initiation factors *EIF5AL1* and *EIF4E1B*, and elongation factor *EEF1E1* (Additional file 7). On the other hand, the cardiac elongation factor *EEF1A2* [34] was up-regulated on D15 (Additional file 7).

Moreover, to explore if non-differentially expressed genes could have been affected by the variation in polysome occupancy (recruitment vs. dissociation), we also compared the polysome/ribosome-free RPKM ratio between hESCs (D0) and cardiomyocytes (D15) (Additional file 6). Interestingly, genes related to translation processes also showed polysomal dissociation on D15 when compared to D0, illustrated by the decreased polysome/ribosome-free ratio (Fig. 7d and e). Among them, there are translation initiation factors (*EIF4A3, EIF4E, EIF4B*), ribosomal proteins (*RPL6, RPL1*4) and RNA helicases (*DDX52*), whose ratios are plotted in Fig. 7f (Additional file 6). Some of these observations were confirmed by qPCR (Additional file 2: Figure S9).

To further confirm that the down-regulation of translation-related genes after cardiomyogenic differentiation could affect protein synthesis, we performed a protein synthesis quantification assay. Cells on D0 (hESC) and D15 (cardiomyocytes) were treated with O-propargyl-puromycin (OPP) which is incorporated into newly translated proteins and then fluorescently labeled. Quantification of fluorescence intensity showed a decrease in protein synthesis after cardiac commitment compared to undifferentiated cells (Fig. 7g and h). Taken together, these findings suggest a translation adjustment during hESC-to-cardiomyocyte differentiation.

Interestingly, other crucial cellular processes also showed regulation by post-transcriptional mechanisms. For instance, on D1 down-buffered and D15 up-buffered GO analysis, the Reactome pathway terms related to cellular metabolism were enriched, suggesting an important post-transcriptional regulation of this process during cardiomyogenic differentiation. Metabolic properties differ between cardiomyocytes and hESCs [35], thus, to understand the regulation of metabolic genes, we grouped the genes annotated as the Reactome pathway terms related to cellular metabolism and called them “Metabolism-related genes” (Additional file 2: Figure S10A and Additional file 7). Comparing the ribosome-free and polysome-bound data, 494 genes were up-regulated on D15, among which 243 (49.2%) were up-loaded, 46 (9.3%) were up-buffered and 205 (41.5%) were up coordinated (Additional file 2: Figure S10B). This likely reflects the post-transcriptional contribution to cardiomyocyte metabolic remodeling demonstrated by metabolic gene recruitment to polysomes.

## Discussion

In this report, we differentiated hESC to cardiomyocytes and analyzed distinct time-points during this process to assess the temporal cell fate transition. The polysome profiling approach followed by ribosome-free and polysome-bound RNA-seq allowed us to evaluate gene regulation during the cardiogenic commitment. Polysome profiling analysis has been used as a robust method to assess the association of ribosomes with mRNAs, providing information about their translational status [23–25]. Here, we show that polysome-bound RNAs reflect the cardiac commitment phenotype, illustrated by down-regulation of pluripotency core regulatory circuitry (*OCT4, SOX2* and *NANOG*) [36] followed by up-regulation of cardiomyogenesis-related genes. The recapitulation of developmental steps is a powerful strategy to control a specific cell fate [1], where the first step is the transition into one of the three embryonic germ layers. The heart originates from the mesoderm emerging from the primitive streak [30]. Mesodermal development genes were found to be strongly regulated on D4, which represents the cardiac mesoderm commitment time-point. The massive number of DEGs between D4 and D9, added to the up-regulation of developmental pathways and pattern specification genes on D4 followed by their down-regulation at progenitor specification stage (D9) are consistent with the complexity of multiple mesodermal lineage choices, recently mapped by Loh et al. (2016) [37].

Moreover, we showed that 60–80% of DEG along cardiomyogenic differentiation were under some degree of post-transcriptional regulation. Uncoupling between the transcriptome and translatome changes, characterized by the independency of the machineries responsible for mRNA availability and engagement in translation, has been observed in most cell types [38]. Here, we observed that many metabolic and cellular processes are regulated exclusively at the level of polysomal association, probably because of post-transcriptional regulatory mechanisms in cellular development [25, 39, 40]. Surprisingly, genes with coordinated regulation (additive regulation) represent a minority of DEGs. In addition to that, changes in the abundance of mRNAs do not directly influence their polysomal engagement, represented by the buffered expression genes. The mRNA access to the translation machinery is governed by the interaction of translation factors with its sequence and structural motifs and is mediated by a complex network of trans-acting proteins and regulatory RNAs [41, 42]. While mRNA levels in the cytoplasm can randomly fluctuate due to transcriptional bursts, the final gene expression levels are adjusted at the time of protein synthesis [43]. McManus et al. (2013) [44] showed that buffering effects on gene expression (translation efficiency opposite to mRNA abundance) were common and would reduce divergent interspecies expression at the protein level. Translational regulation has already been reported in mouse mesoderm commitment [45] and hESC neuronal differentiation [46]. Here, we show unprecedented data about post-transcriptional regulation during human cardiomyogenic commitment.

We also found that the translational activity is reduced during differentiation; this became more evident when comparing hESC on D0 to cardiomyocytes on D15. Protein synthesis rate in cardiac tissue decreases from fetal to adult development, and in adult heart is lower than in other tissues [47, 48]. Chorghade et al. (2017) showed that the translation initiation RNA-binding protein PABPC1 is post-transcriptionally down-regulated in the adult heart tissue, which possibly decreases the translational capacity of the heart. Our data showed that the D15 down-regulated genes were enriched in RNA processing and translation genes, which were mostly down-regulated only in the polysome-bound fraction. Translation-related genes also showed polysomal dissociation on D15 when compared to D0, illustrated by the decreased polysome/ribosome-free ratio, further suggesting a strong post-transcriptional regulation and corroborating previous observations [38, 49, 50].

Protein synthesis is an indispensable process for maintaining homeostasis in the cell, and aberrations in its regulation may contribute to a wide range of diseases [51]. We have previously investigated the translational regulation during cell commitment to adipogenesis in hASCs. Our results showed the translational control as a key mechanism regulating the early steps of adipogenic differentiation, with a significant reduction in protein synthesis and a lower translational efficiency of ribosomal proteins [52]. In vitro differentiation of other cell types also showed decreasing translation rate, due to, e.g., eIF2a phosphorylation or rRNA biogenesis [53–57]. Blair et al. (2017) reported the translational down-regulation of translation-related genes after hESC neuronal differentiation (including translation initiation factors and ribosomal proteins) [46], indicating that this mechanism doesn’t seem to be cardiac-specific. In contrast, Blanco et al. (2016) showed that adult stem cells have lower protein synthesis rates than committed cells, using skin as a model. In normal skin, the RNA methyltransferase NSUN2 expression is restricted to committed hair follicle populations. They have demonstrated that loss of NSUN2 causes hypomethylation of tRNAs, accumulation of 5′ tRNA fragments, which repress cap-dependent protein translation [58–60]. This reduced translation in adult stem cells is probably related to the biology of these specific cell types. Adult stem and progenitor cells are metabolically quiescent and low, if at all, proliferative. After stimulation by injury, they enter an activation state where they proliferate and show increased translational rates [61]. These particular characteristics make the comparison of the regulatory pathways and mechanisms underlying translational regulation in adult and embryonic stem cells not possible as these cells are in different stages of differentiation and in completely different biological environments. Our model recapitulates embryonic differentiation, where highly proliferative stem cells commit and differentiate into cell types with defined gene expression patterns and, in most cases, lower proliferative rates.

Noncontractile stem cells require less energy than beating cardiomyocytes, therefore, a transition in energetic infrastructure is necessary to support the increased energetic needs during cardiac differentiation [62, 63]. This switch in energy metabolism is associated with increased mitochondrial maturation and oxygen consumption and reduced glycolysis, due to a metabolic transcriptome remodeling [35, 62]. We showed that the metabolic reconfiguration is also a consequence of post-transcriptional level gene regulation. Almost 50% of metabolism-related genes regulated on D15 were increased only in polysome-bound fraction, characterizing the higher recruitment of these genes to the translational machinery. Increasing evidence has been showing the role of translational control in regulating metabolic function and that its defect is implicated in the pathogenesis of metabolic disorders [64, 65]. Additionally, cardiomyocyte mRNA interactome revealed that metabolic enzymes can act as RBPs [66], and energy metabolism proteins were found associated with ribosomes in ESCs, for instance, controlling the translation of mRNAs [67]. All these lines of evidence suggest a close relationship between cellular metabolism and translational control which deserves further exploration.

## Conclusions

Polysome profiling followed by ribosome-free and polysome-bound RNA-seq of hESC cardiogenic commitment allowed us to evaluate gene expression regulation, revealing a massive control of developmental steps. Post-transcriptional regulation might be acting on 60–80% of DEG along cardiomyogenic differentiation, contributing to the final phenotype. Cardiomyocytes translational activity was reduced when compared to undifferentiated cells showing that translation regulation is also controlled during differentiation. Understanding the biological processes that trigger differentiation is crucial for an improvement of in vitro differentiation strategies and discovery of endogenous regenerative pathways. Our results helped to uncover transcriptional and post-transcriptional regulation involved in cardiomyogenic commitment gene expression, collected unprecedented data regarding polysome-bound RNAs and provided advances in comprehension of differentiation processes. Altogether, this work could prospect fundamental tools to develop new therapy and research approaches.

## Methods

### Cell culture and cardiomyocyte differentiation

hES-NKX2–5^eGFP/w^ cell line was a gift from Dr. David A. Elliott (Monash Immunology and Stem Cell Laboratories, Monash University, Australia) [27]. Cells were maintained on irradiated MEFs (mouse embryonic fibroblasts) using DMEM-F12 (Gibco™) with 20% KSR (Gibco™), 0.1 mM non-essential amino acids, 55 μM β-mercaptoethanol, 100 μg/mL penicillin-streptomycin, 2 mM L-glutamine and 10 ng/mL bFGF. The cardiomyocyte differentiation protocol was adapted from the previously published one [2]. Seventy-two hr. before differentiation, 7 × 10^5^ cells/well were passaged onto a 6-well plate coated with Growth Factor Reduced BD® Matrigel Matrix for feeder depletion. Then, cells were dissociated with collagenase I (1 mg/mL) for 20 min followed by trypsin-EDTA (0.05%) for approximately 30 s and scraped to form small clusters. After washing, the clusters were cultured in 6-well Ultralow Attachment Corning plates using StemPro-34 (Invitrogen), supplemented with 100 μg/ml penicillin-streptomycin, 2 mM L-glutamine, 150 μg/mL transferrin, 50 μg/mL ascorbic acid and 0.45 mM monothioglycerol (MTG) and kept in humid incubator at 37 °C, 5% CO_2_ and 5% O_2_. For embryoid bodies (EBs) aggregation (D0-D1), the basal medium was supplemented with 1 ng/mL BMP4. On D1, the medium was replaced with supplementation of 10 ng/mL BMP4, 6 ng/mL Activin A and 5 ng/mL bFGF; on D4 with 10 μg/mL VEGF and 10 μM XAV 939, and starting on D8, the medium was replaced every two or three days supplemented with 10 μg/mL VEGF and 1 ng/mL BMP4. Three independent differentiation assays were used as experimental replicates.

### Flow cytometry

EBs were dissociated on D4 using trypsin-EDTA (0.05%) and incubated with the surface marker PE-conjugated anti-CD56 (1:25 in 0.5% PBS/BSA, BD cat. 347,747) and 1 μg/μL DAPI. On D9, cells were disaggregated with trypsin-EDTA (0.05%) for 5 min and resuspended in PBS to evaluate eGFP expression. On D15, EBs were disaggregated using 1 mg/mL collagenase I for 16 h and trypsin-EDTA (0.05%) for 5 min, fixed with 4% paraformaldehyde, permeabilized with 0.5% Triton X-100 and incubated with anti-troponin T antibody (1:100 in 0.5% PBS/BSA, cardiac isoform Ab-1, Thermo Scientific™, cat. #MS-295-P0) followed by Pacific Blue-conjugated anti-mouse antibody (11000). Analyses were carried out using a FACSCanto II flow cytometer and FlowJo software.

### Immunofuorescence and fluorescent microscopy

On D15, EBs were disaggregated using 1 mg/mL collagenase I for 16 h and trypsin-EDTA (0.05%) for 5 min and plated on Matrigel-coated wells. After 2–5 days, cardiomyocytes were fixed with 4% paraformaldehyde, permeabilized and blocked with 0.5% Triton X-100 and 1% PBS-BSA. Overnight incubation with anti-troponin I antibody (1:100 in 0.5% PBS/BSA, Santa Cruz Biotechnology, cat.: sc-15,368) followed by Alexa 546-conjugated anti-rabbit IgG (1:800, Invitrogen) and 1 μg/μL DAPI. EBs or fixed cardiomyocytes were visualized using a Leica DMI6000B optical microscope and images and videos acquired by LAS AF software.

### Polysome profile and RNA isolation

At the indicated time points, monolayer hESC on D0 or differentiating EBs were treated with 0.1 mg/mL cycloheximide (Sigma-Aldrich) for 10 min at 37 °C, disaggregated with trypsin-EDTA (0.05%) for 10 min and washed twice with PBS. Cells were resuspended in polysome lysis buffer (15 mM Tris HCl, pH 7.4, 15 mM MgCl2, 300 mM NaCl, 1% Triton X-100, 40 U/μL RNAse Out, 24 U/mL DNAse and 100 μg/mL cycloheximide), incubated for 10 min on ice and centrifuged at 12000 x g for 10 min at 4 °C. For the puromycin control, cells were treated with 2 mg/mL puromycin for 1 h before disaggregated and the buffers were used without cycloheximide. The supernatants were loaded onto 10 to 50% sucrose gradients (prepared with BioComp model 108 Gradient Master) and centrifuged at 150000 x g (SW40 rotor, HIMAC CP80WX HITACHI) for 160 min at 4 °C. Different sucrose gradient fractions were separated using ISCO gradient fractionation system (ISCO Model 160 Gradient Former Foxy Jr. Fraction Collector), connected to a UV detector, which monitored the absorbance at 275 nm to record the polysome profile. RNA from ribosome-free and pooled polysomal fractions was isolated using the Direct-zol RNA MiniPrep (Zymo Research), following the manufacturer’s instructions.

### High-throughput sequencing and data analysis

For cDNA library preparation, 200 to 500 ng of ribosome-free or 2 μg of polysome-bound RNA were used to perform three independent sample replicates. The cDNA libraries were prepared using the TruSeq Stranded mRNA Sample Preparation kit (Illumina, Inc.), and RNA-seq was carried out in an Illumina HiSeq platform. Mapping and counting of sequencing data was performed with the Rsubread package [68] against the new version of the human genome GRCh38. Mapping parameters were set for unique mapping of the reads (the rest were default). For some calculations, CPM values (counts per million) were determined, where each sample was normalized to one million reads to account for the library size (Additional file 8).

To assess the quality of the experiment and reproducibility of results, we performed a correspondence analysis (COA), a dimension reduction method of the matrix of counts. In COA, it is possible to simultaneously visualize samples and genes, revealing associations between them.

Differential expression analysis was done using the Bioconductor R package edgeR [69]. Several comparisons were performed for both ribosome-free and polysome-bound RNA fractions - each sample against the preceding time-point: D0 vs D1, D1 vs D4, D4 vs D9 and D9 vs D15; and D0 vs D15. For these analyses, we retained only those genes with at least one count per million in at least three samples. After a normalization procedure using three recommended methods (estimateGLMCommonDisp, estimateGLMTrendedDisp, estimateGLMTagwiseDisp), differential expression analysis for all 10 comparisons was performed using the generalized linear mixed model (glmFit and glmLRT). Correction for multiple testing was performed with FDR and RPKM values for each sample were also determined to compare the expression between the samples (Additional file 3, Additional file 4 and Additional file 7).

Clustering was performed with the k-means algorithm on the table of read log-counts of each gene. Biological replicates were averaged (mean of 3 replicates) and only differentially expressed genes in either condition (D0 vsD1, D1 vs D4, D4vs D9, D9 vs D15) were considered. Different numbers of k were tested by measuring the within sum of squares. After visual inspection of within sum of squares distribution, we decided on k = 9.

Polysome/ribosome-free ratio was determined by dividing RPKM values derived from the polysomal fraction by the ones derived from the ribosome-free experiment (three biological replicates were averaged).

Gene ontology (GO) analysis was performed using Enrich R (http://amp.pharm.mssm.edu/Enrichr/).

### Protein synthesis rate analysis

Protein synthesis was measured using Click- iT® Plus OPP Protein Synthesis Assay (Molecular Probes, Grand Island, NY). Cells on D0 (hESC) or D15 (cardiomyocytes) were seeded in matrigel-coated 96-well plates, and after 48 h, the staining and detection was performed following the manufacturer’s instructions. The quantitative analysis was performed using an Operetta HTS imaging system (PerkinElmer, Waltham MA, USA). Images of 25 fields per well were evaluated with Harmony 3.5.2 software (PerkinElmer). Fluorescence intensities were measured at the cytoplasm regions around the nucleus. For each condition, 1400 cells were randomly chosen for intensity analysis.

## Additional files

Additional file 1: Video of beating D15 EBs. (MP4 1974 kb)

Additional file 2: **Figure S1.** Summary of high-throughput ribosome-free and polysome-bound RNA-seq of distinct cardiomyogenic differentiation time-points (*n* = 3). **Figure S2.** (A) Polysome profiling of D15 cells treated with cycloheximide or puromycin. Ribosome-free (fractions 1–3), monosome (fractions 5–7), light polysomes (fractions 9–15) and heavy polysomes (fractions 16–22) fractions were pooled and isolated. (B) Cardiomyocyte markers evaluated by qPCR on distinct polysome fractions. **Figure S3.** Polysome-bound validation of developmental markers expression using qPCR. **Figure S4.** (A) Number of differentially expressed genes on each differentiation time-point, compared to previous time-point (FDR < 0.05, − 2 > logFC> 2) on ribosome-free samples. Number of protein-coding and non-coding genes are also shown (bottom panel). (B) Non-coding genes categories of DEGs (all time-points combined, each time-point against its previous for analysis) (FDR < 0.05, − 2 > logFC> 2) on polysome-bound samples. **Figure S5.** Gene Ontology EnrichR BP enriched terms for up (FDR < 0.05, logFC> 2) and down (FDR < 0.05, logFC<− 2) polysome-bound regulated genes. **Figure S6.** Genes coordinately regulated are under control of transcriptional and post-transcriptional regulation during cardiomyogenic differentiation. **Figure S7.** qPCR validation of DEG during cardiomyogenic differentiation. **Figure S8.** Non-DEGs showed differences on polysome recruitment and dissociation on D0 vs. D1 and D9 vs. D15. **Figure S9.** RNA related-genes validation by qPCR. **Figure S10.** Cardiomyocytes (D15) showed up-regulation of cellular metabolism genes. (DOCX 2575 kb)

Additional file 3: Polysome-bound RNA-seq data of comparisons of each sample against the preceding time-point (FDR < 0.05). (XLSX 5476 kb)

Additional file 4: Ribosome-free RNA-seq data of comparisons of each sample against the preceding time-point (FDR < 0.05). (XLSX 1508 kb)

Additional file 5: Polysome-bound vs. ribosome-free analysis. (XLSX 41 kb)

Additional file 6: Polysome-bound vs. ribosome-free ratios (FDR < 0.05). (XLSX 17874 kb)

Additional file 7: Polysome-bound and ribosome-free RNA-seq data of D0 vs. D15 comparison (FDR < 0.05). (XLSX 3849 kb)

Additional file 8: Counts and RPKM values of all mapped genes. (XLSX 30119 kb)

## Abbreviations

BP: Biological process
COA: Correspondence analysis
CPM: Counts per million mapped reads
DEG: Differentially expressed genes
EB: Embryoid body
FC: Fold change
FDR: False discovery rate
GO: Gene Ontology
hASCs: Human adipose stem cells
hESC: Human embryonic stem-cell
OPP: O-propargyl-puromycin
RPKM: Reads per kilobase per million mapped reads
