# Supplementary material for: Cardiomyogenic differentiation is fine-tuned by differential mRNA association with polysomes
Source: BMC Genomics. 2019 Mar 15;20:219. doi: 10.1186/s12864-019-5550-3 (PMC6420765; doi:10.1186/s12864-019-5550-3)
Supplement: Supplementary file 2 — Figure S1. Summary of high-throughput ribosome-free and polysome-bound RNA-seq of distinct cardiomyogenic differentiation time-points (n = 3). Figure S2. (A) Polysome profiling of D15 cells treated with cycloheximide or puromycin. Ribosome-free (fractions 1–3), monosome (fractions 5–7), light polysomes (fractions 9–15) and heavy polysomes (fractions 16–22) fractions were pooled and isolated. (B) Cardiomyocyte markers evaluated by qPCR on distinct polysome fractions. Figure S3. Polysome-bound validation of developmental markers expression using qPCR. Figure S4. (A) Number of differentially expressed genes on each differentiation time-point, compared to previous time-point (FDR < 0.05, − 2 > logFC> 2) on ribosome-free samples. Number of protein-coding and non-coding genes are also shown (bottom panel). (B) Non-coding genes categories of DEGs (all time-points combined, each time-point against its previous for analysis) (FDR < 0.05, − 2 > logFC> 2) on polysome-bound samples. Figure S5. Gene Ontology EnrichR BP enriched terms for up (FDR < 0.05, logFC> 2) and down (FDR < 0.05, logFC<− 2) polysome-bound regulated genes. Figure S6. Genes coordinately regulated are under control of transcriptional and post-transcriptional regulation during cardiomyogenic differentiation. Figure S7. qPCR validation of DEG during cardiomyogenic differentiation. Figure S8. Non-DEGs showed differences on polysome recruitment and dissociation on D0 vs. D1 and D9 vs. D15. Figure S9. RNA related-genes validation by qPCR. Figure S10. Cardiomyocytes (D15) showed up-regulation of cellular metabolism genes. (DOCX 2575 kb) [file 12864_2019_5550_MOESM2_ESM.docx]

Pereira et al., 2018

**Cardiomyogenic differentiation is fine-tuned by differential mRNA association with polysomes.**

**Supplemental Figures**


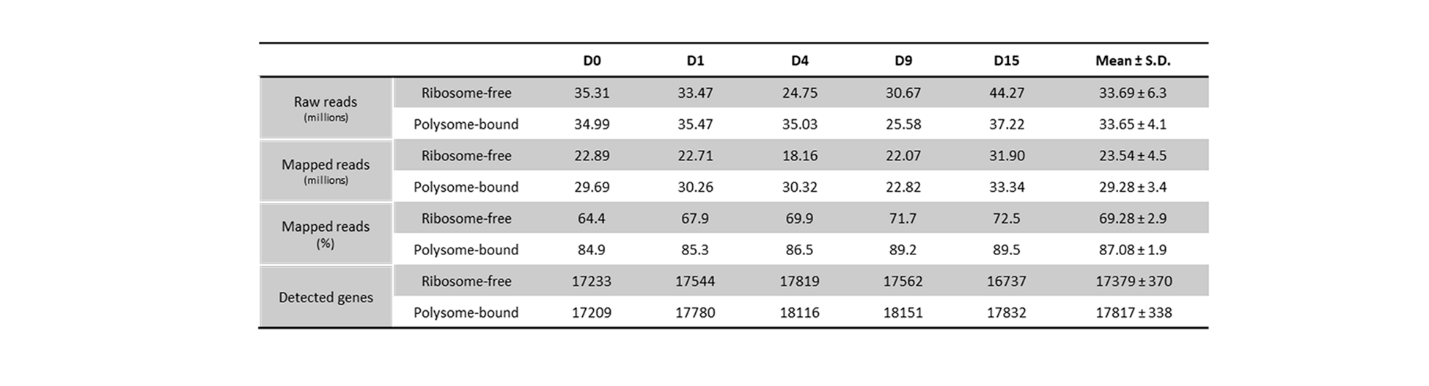


**Figure S1.** Summary of high-throughput ribosome-free and polysome-bound RNA-seq of distinct cardiomyogenic differentiation time-points (n=3). Related to Figure 1.


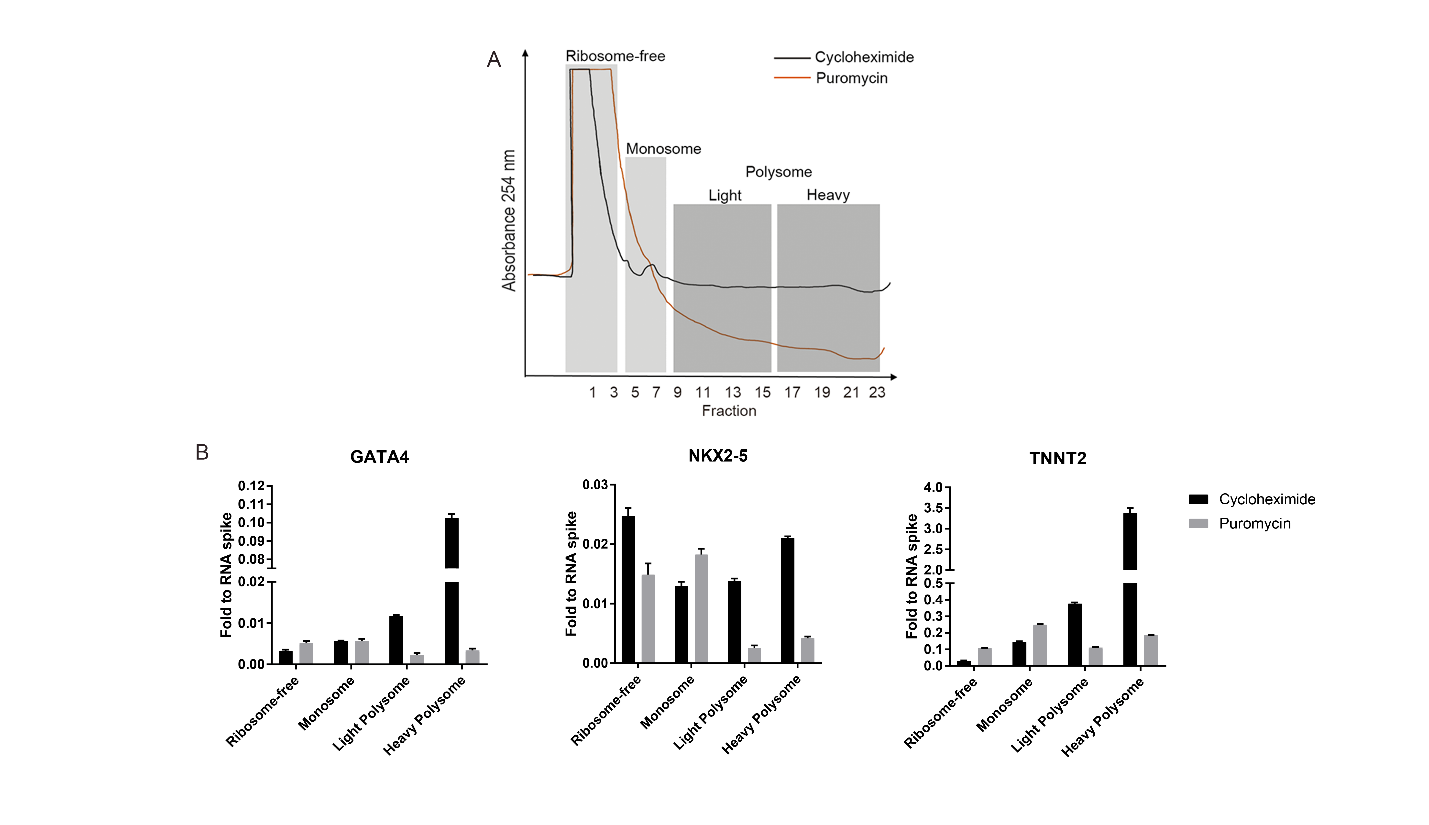


**Figure S2**. (A) Polysome profiling of D15 cells treated with cycloheximide or puromycin. Ribosome-free (fractions 1-3), monosome (fractions 5-7), light polysomes (fractions 9-15) and heavy polysomes (fractions 16-22) fractions were pooled and isolated. (B) Cardiomyocyte markers evaluated by qPCR on distinct polysome fractions.

**
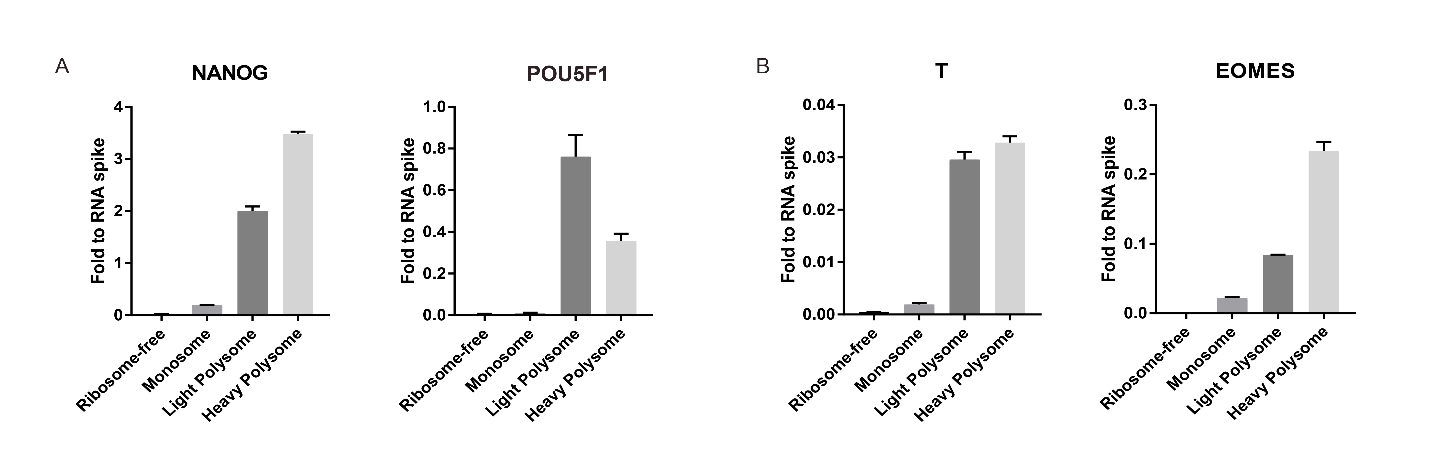
**

**Figure S3**. Polysome-bound validation of developmental markers expression using qPCR. (A) NANOG and POU5F1 showed polysome association on D0 and (B) T and EOMES on D4 of cardiomyogenic differentiation.


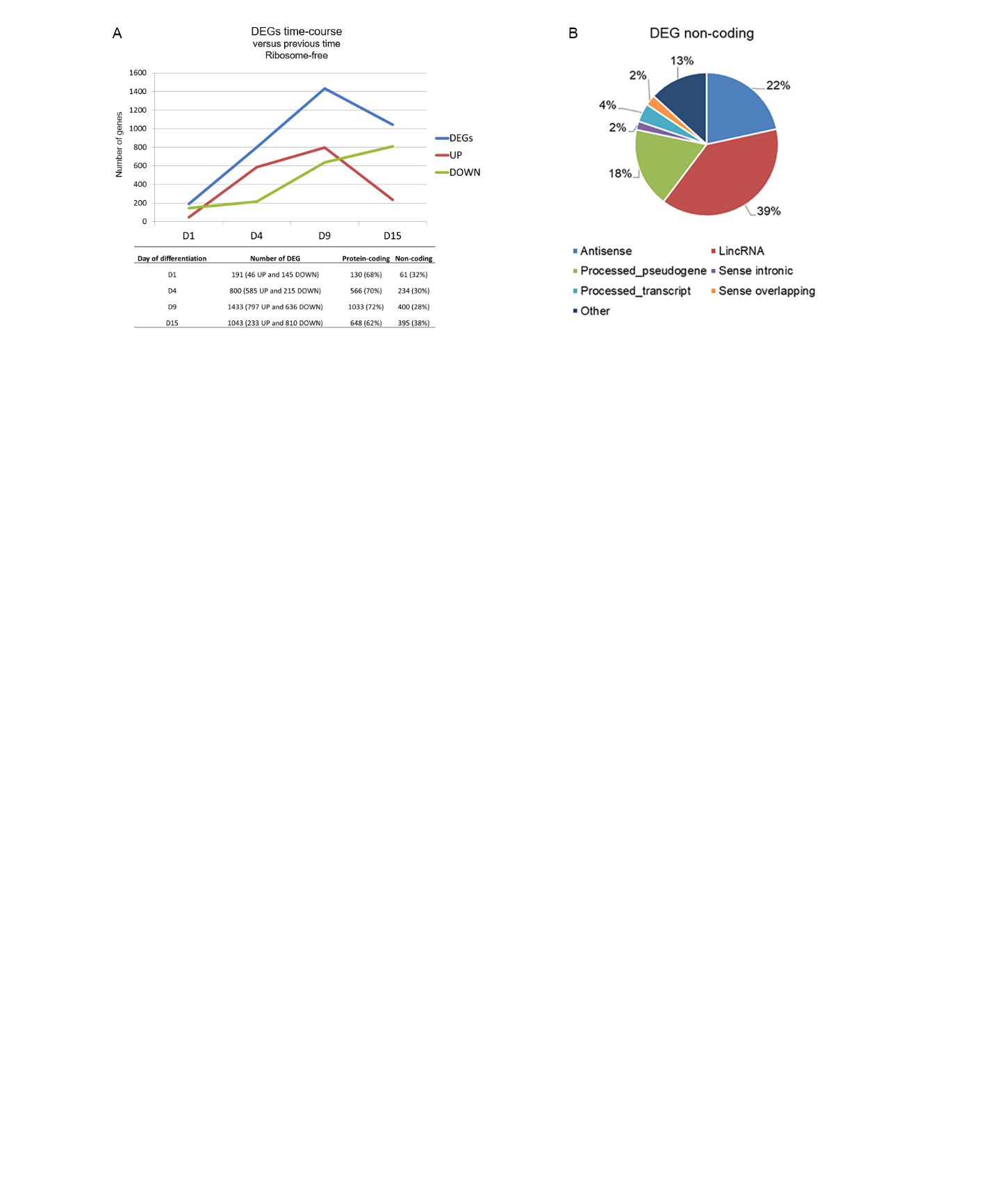


**Figure S4.** (A) Number of differentially expressed genes on each differentiation time-point, compared to previous time-point (FDR<0.05, -2>logFC>2) on ribosome-free samples. Number of protein-coding and non-coding genes are also shown (bottom panel). (B) Non-coding genes categories of DEGs (all time-points combined, each time-point against its previous for analysis) (FDR<0.05, -2>logFC>2) on polysome-bound samples. Related to Figure 2.


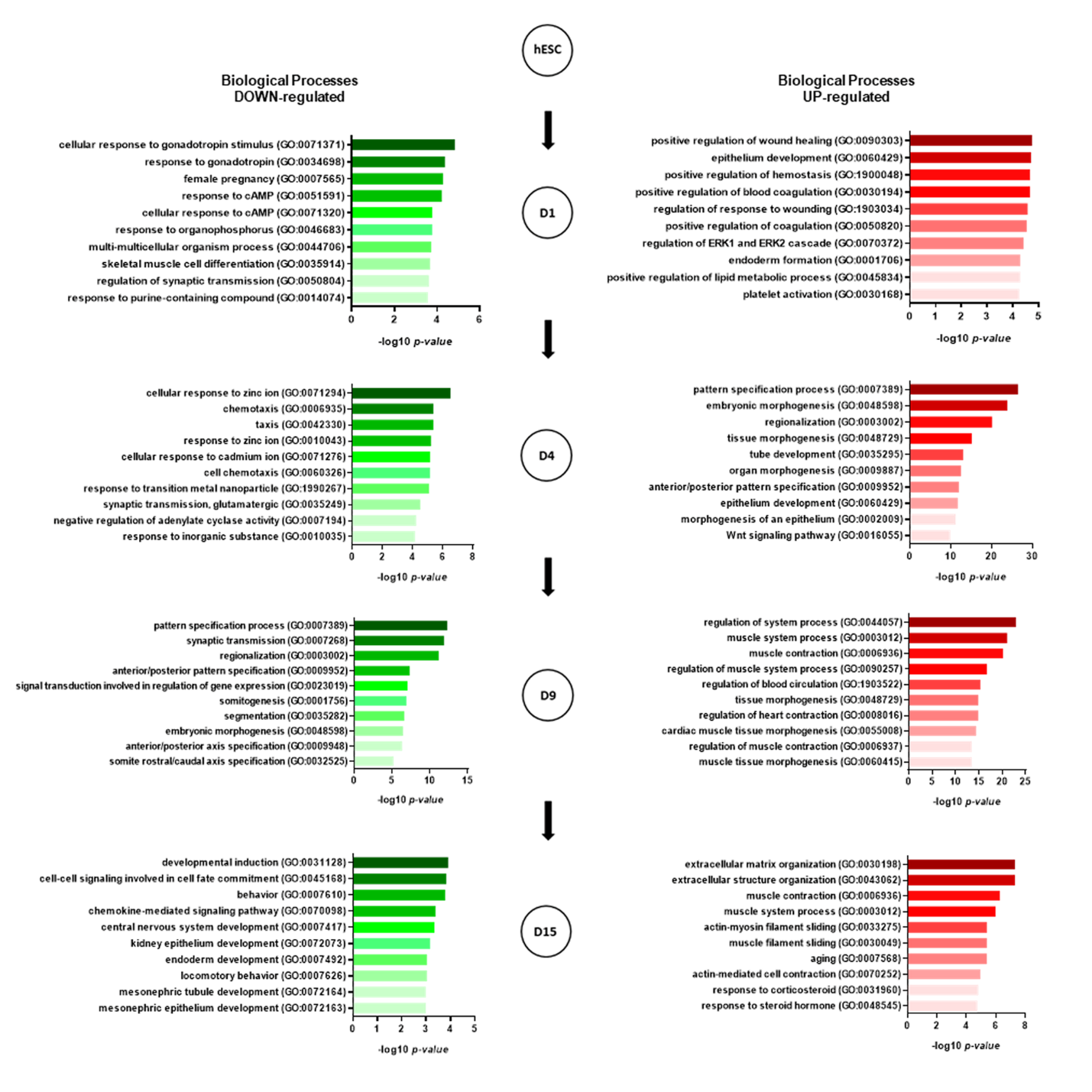


**Figure S5.** Gene Ontology EnrichR BP enriched terms for up (FDR<0.05, logFC>2) and down (FDR<0.05, logFC<-2) polysome-bound regulated genes. 10 terms with lower p-value are shown. Related to Figure 2.


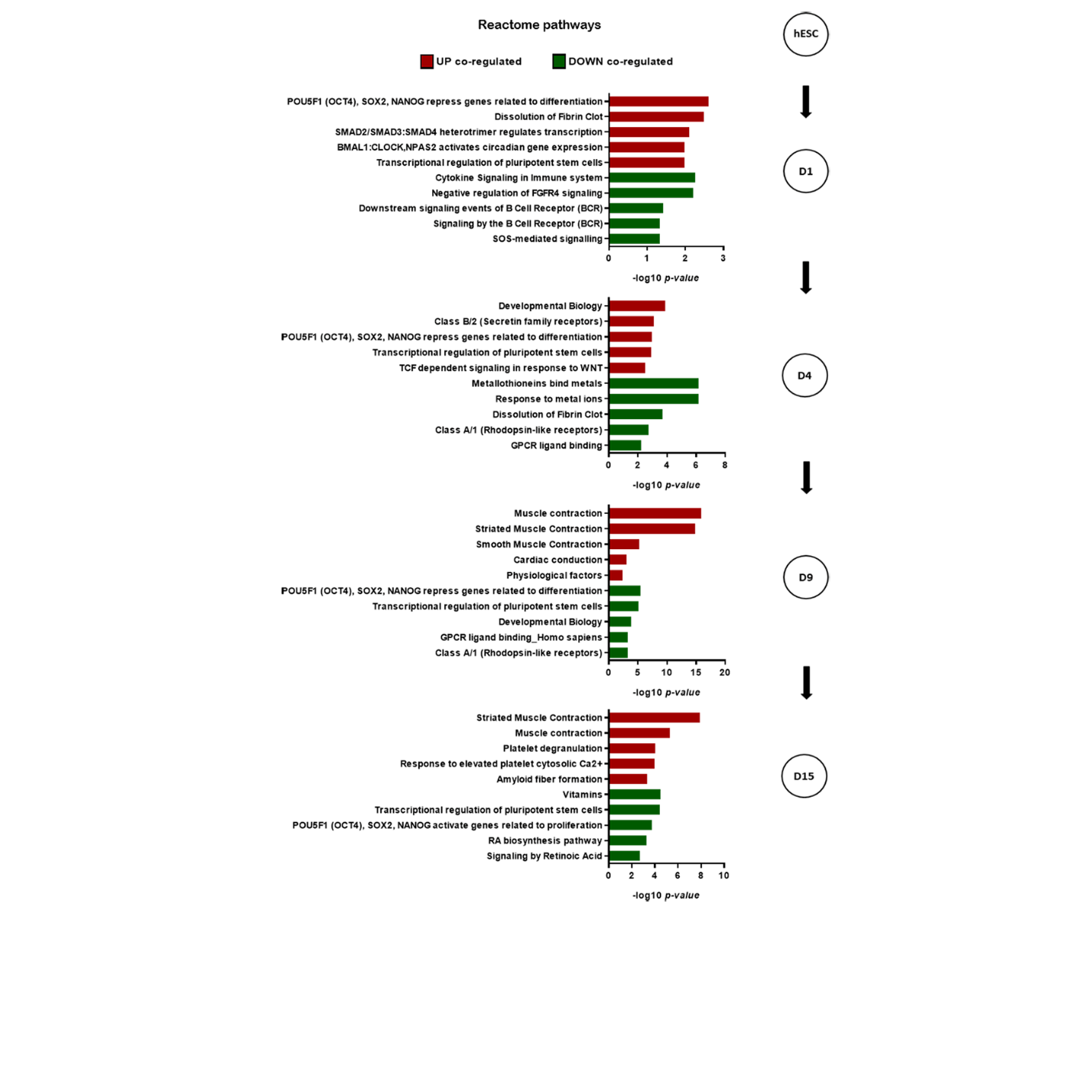


**Figure S6.** Genes coordinately regulated are under control of transcriptional and post-transcriptional regulation during cardiomyogenic differentiation. GO Reactome pathways enriched terms for up (FDR<0.05, logFC>2, RPKM>1) and down (FDR<0.05, logFC<-2, RPKM>1) co-regulated genes. 5 terms with lower p-value are shown. Related to Figure 4.


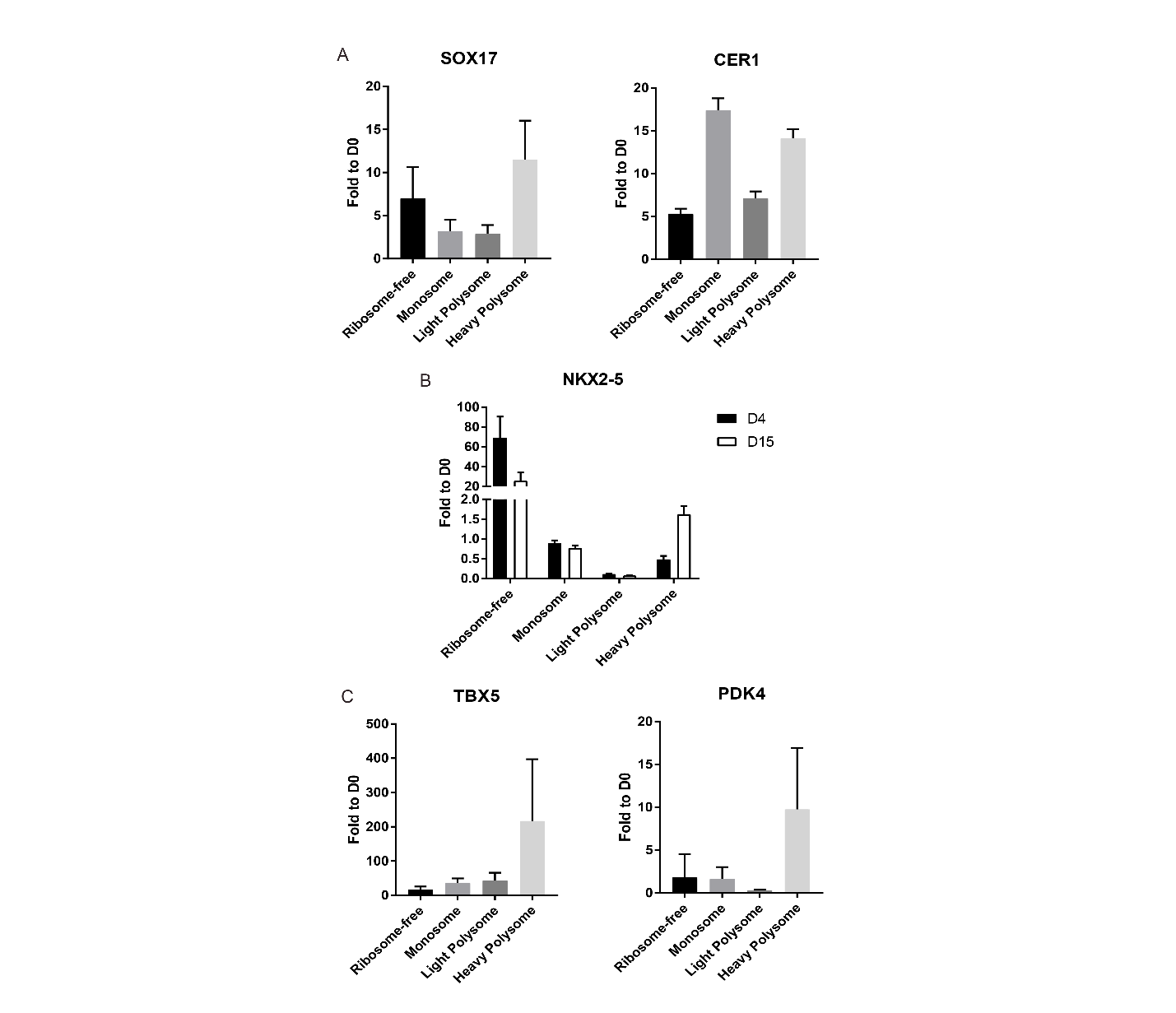


**Figure S7**. qPCR validation of DEG during cardiomyogenic differentiation. (A) On D4, CER1 and SOX17 showed up-regulated on polysome-bound fraction. (B) NKX2-5 showed an interesting post-transcription regulation on D4 and D15. (C) On D15, TBX5 and PDK4 showed up-regulated on polysome-bound fraction.


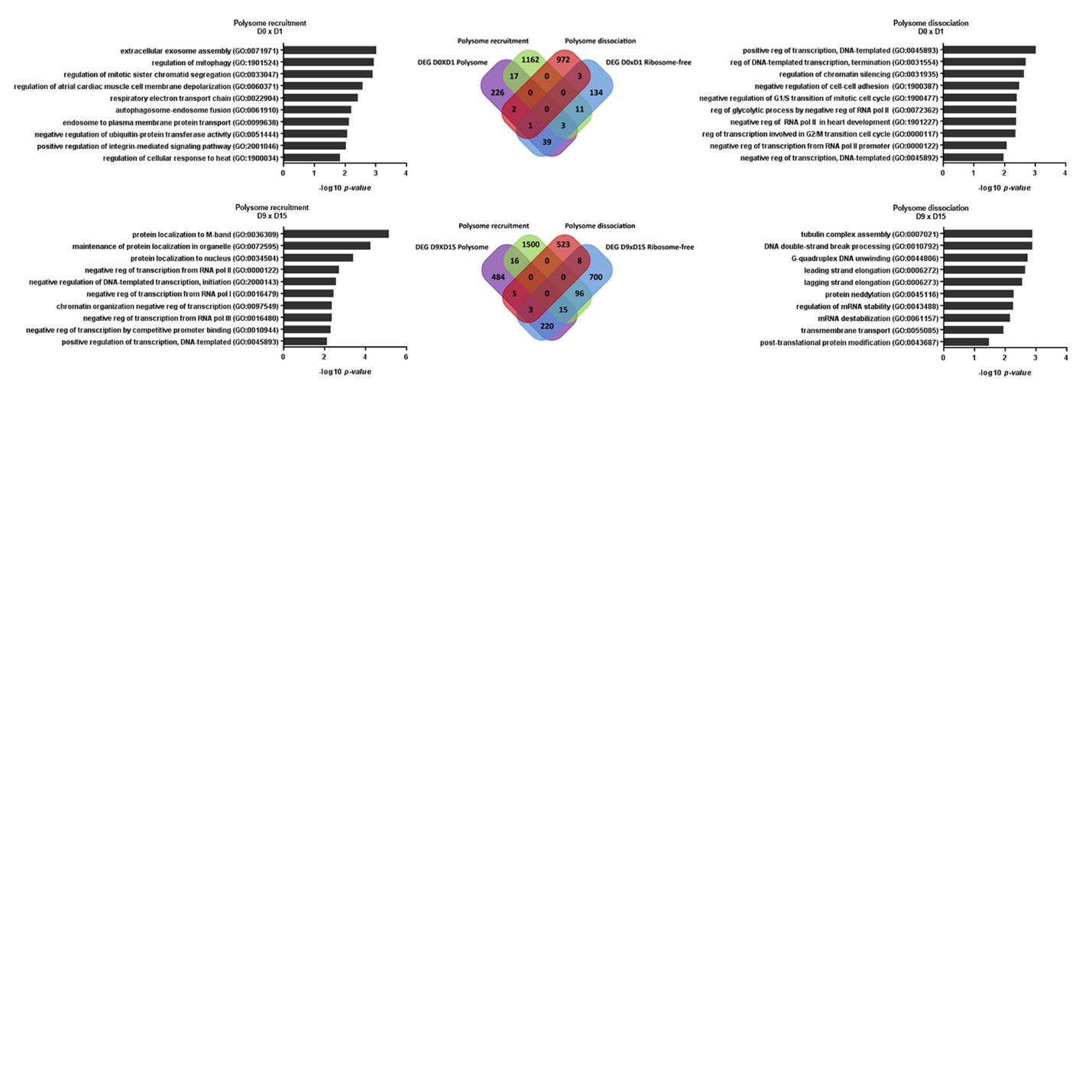


**Figure S8.** Non-DEGs showed differences on polysome recruitment and dissociation on D0 vs. D1 and D9 vs. D15. Venn diagram and EnrichR BP enriched terms of polysome recruitment (FDR<0.05, logFC>2) or dissociation (FDR<0.05, logFC<-2) non-DEG based on polysome/ribosome-free ratio. Related to Figure 6.


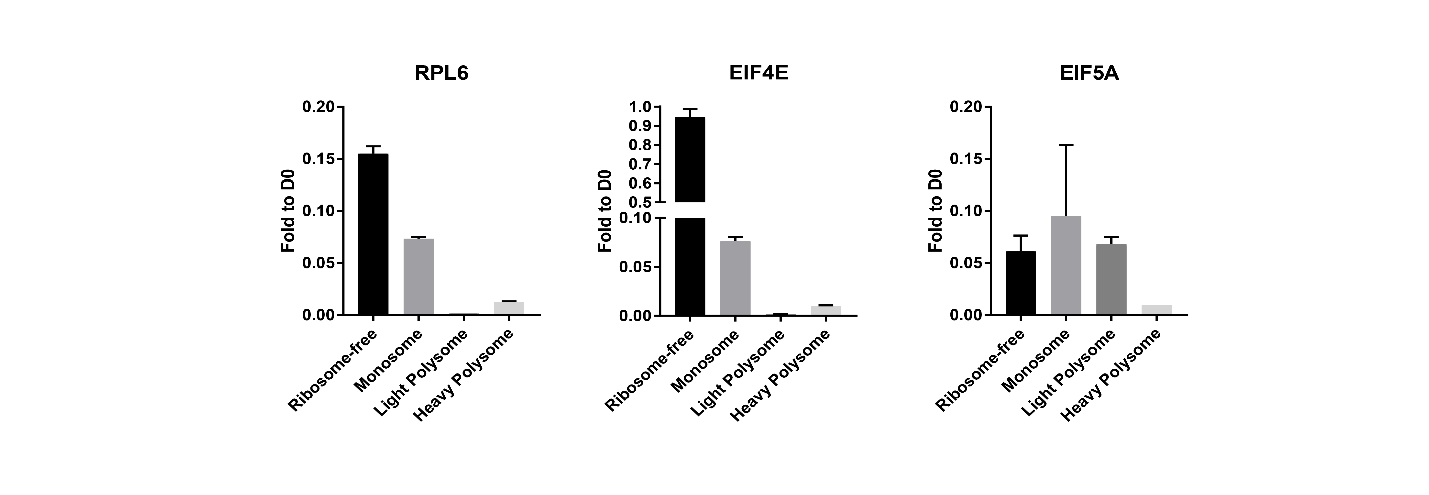


**Figure S9**. RNA related-genes validation by qPCR. RPL6, EIF5A and EIF4E differential association with polysomes on D15 when comparing to D0.


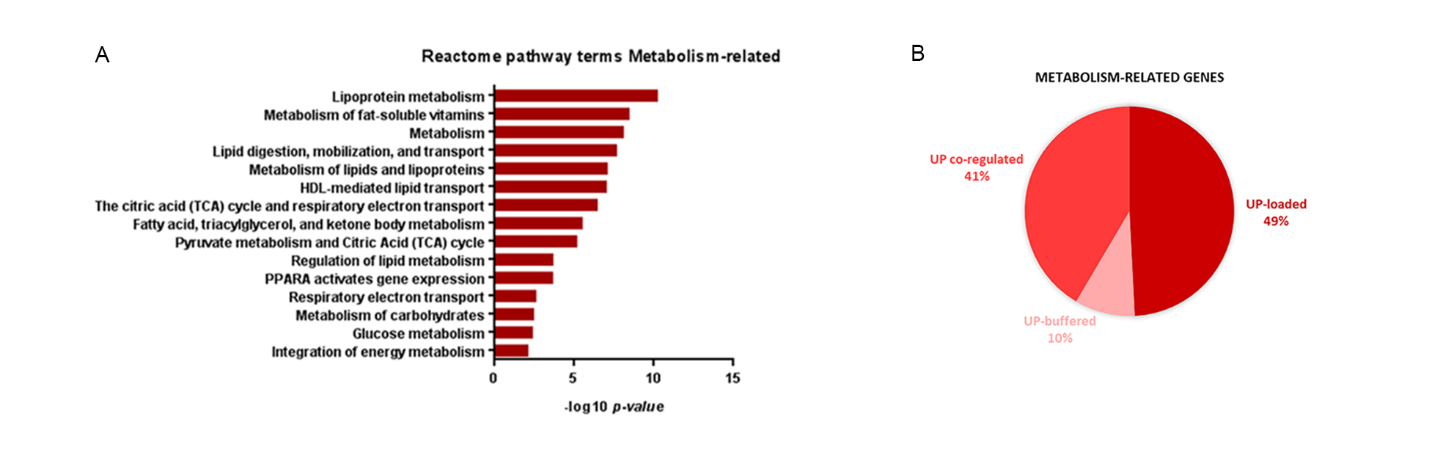


**Figure S10.** Cardiomyocytes (D15) showed up-regulation of cellular metabolism genes. (A) GO Reactome pathways terms enriched for D15 up-regulated genes (FDR<0.05, logFC>1) when compared to hESC (D0). (B) Genes classified on Metabolism-related Reactome pathways (A) were transcriptional and post-transcriptionally regulated according to the co-regulated, buffered and loaded classification as indicated.
